# Natural Variation in Petal Color in *Lycoris longituba* Revealed by Anthocyanin Components

**DOI:** 10.1371/journal.pone.0022098

**Published:** 2011-08-04

**Authors:** Qiuling He, Ye Shen, Mingxiu Wang, Minren Huang, Ruizhen Yang, Shuijin Zhu, Liangsheng Wang, Yanjun Xu, Rongling Wu

**Affiliations:** 1 College of Forest Resources and Environment, Nanjing Forestry University, Nanjing, People's Republic of China; 2 Department of Agronomy, College of Agriculture and Biotechnology, Zhejiang University, Hangzhou, People's Republic of China; 3 Beijing Botanical Garden, Institute of Botany, Chinese Academy of Sciences, Beijing, People's Republic of China; 4 Department of Applied Chemistry, China Agricultural University, Beijing, People's Republic of China; 5 Center for Computational Biology, Beijing Forestry University, Beijing, People's Republic of China; 6 National Engineering Laboratory for Tree Breeding, Beijing Forestry University, Beijing, People's Republic of China; 7 Key Laboratory of Genetics and Breeding in Forest Trees and Ornamental Plants, Beijing Forestry University, Beijing, People's Republic of China; Purdue University, United States of America

## Abstract

*Lycoris longituba* is one of the species belonging to the Amaryllidaceae family. Despite its limited distribution, endemic to central eastern China, this species displays an exceptionally wide diversity of flower colors from purple, red, orange, to yellow, in nature. We study the natural variation of floral color in *L. longituba* by testing the components of water-soluble vacuolar pigments – anthocyanins – in its petals using high-performance liquid chromatography coupled with photodiode array detection and electrospray ionization mass spectrometry. Four anthocyanins were identified, cyanidin-3-sophoroside (Cy3So), cyanidin-3-xylosylglucoside (Cy3XyGlc), cyanidin-3-sambubioside (Cy3Sa), and pelargonidin-3-xylosylglucoside (Pg3XyGlc), which occur at various amounts in *L. longituba* petals of different colors. A multivariate analysis was used to explore the relationship between pigments and flower color. Anthocyanins have been thought to play a major role in acting as a UV screen that protects the plant's DNA from sunlight damage and attracting insects for the purpose of pollination. Thus, knowledge about the content and type of anthocyanins determining the petal coloration of *Lycoris longituba* will help to study the adaptive evolution of flowers and provide useful information for the ornamental breeding of this species.

## Introduction

**Figure 1:**
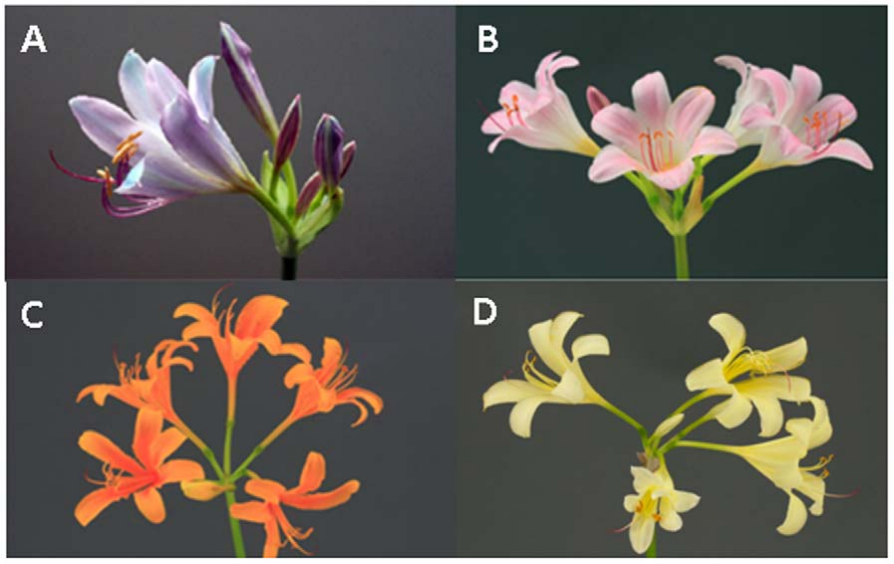
Flowers of four representative colors, purple (A), red (B), orange (C), and yellow (D), for *Lycoris longituba* variants sampled from a natural population.

Endemic in China, *Lycoris longituba* (Y. Hsu et Q.J. Fan), a species from the Amaryllidaceae family, is characterized by a few biological features that are interesting to evolutionary studies. First, this species is naturally distributed in a limited range, including the Langya Mountain of Anhui Province, the Baohua Mountain and Xuyi City of Jiangsu Province, all about 50 km from one another [1], but it displays an incredible diversity of floral colors. As shown in Fig. 1, four distinct representatives of floral colors, purple, red, orange, and yellow, commonly occur in *L. longituba*. Such great variability provides an excellent model system for identifying the biochemical determinants of flower colors and explaining these determinants in terms of plant ecological and evolutionary processes [2]. Second, leaves of *L. longituba* sprout in spring and wither in early summer, followed by flowering. These discrete phases of vegetative growth and reproduction, a rare phenomenon in angiosperms, may offer an unprecedented opportunity to study the evolution of fitness traits in plants. In addition, *L. longituba* has many ornamental and medical values and can be potentially interesting to plant breeding.

As part of the ongoing *Lycoris longituba* evolutionary genetics project, we attempted to study the phytochemical components that form floral colors in this species and further use these components to interpret the mechanism of why there is a diversity of flower colors within such a limited range of distribution. Floral colors are due to biological pigments including a variety of different kinds of molecules, such as porphyrins, carotenoids, anthocyanins and betalains [3]. These biological pigments absorb some wavelengths of light and reflect others. The absorbed light may be used by the plant to perform chemical reactions, while the reflected wavelengths of light determine the color of the flower.

Anthocyanin-type pigments, found only in terrestrial plants, are thought to be evolutionarily adaptive for several different plants processes [4]–[6]. In photosynthetic tissues, such as leaves and sometimes stems, anthocyanins serve as a sunscreen blocking blue-green and UV light, thus protecting cells from excessive light damage and the tissues from photoinhibition or excessive light stress [7], [8]. In flowers, anthocyanins may appear red, purple, or blue, according to pH values [9], [10], which helps in attracting pollinators [11]. In fruits, the colorful skins derived from anthocyanins also attract animals to eat the fruits and, therefore, disperse the seeds [12]. Because of their significant roles in plant function, anthocyanins have been used to study the molecular genetic basis of variation and evolution in morphological traits [11] and potentially used as an indirect trait for plant breeding [6].

There has been a wealth of literature on determining anthocyanin components in the Amaryllidaceae family. For example, four anthocyanin components, pelargonidin-3-glucosides (Pg3G), pelargonidin-3-xylosylglucosides (Pg3XyGlc), cyanidin-3-glucosides (Cy3G), and cyanidin-3-xylosylglucosides (Cy3XyGlc) were identified in *L. radiata* (L'Her.) Herb [13]. In Nerine, a genus of the Amaryllidaceae family, Arisumi and Shioya [14] detected Cy3G, Pg3G, cyanidin-3,5-diglucoside (Cy3G5G), peonidin-3,5-diglucoside (Pn3G5G), pelargonidin- 3,5-diglucoside (Pg3G5G), and cyanidin-3-sophoroside (Cy3So). In the flowers of *Hippeastrum*, cyaniding-3-*O*-(6″-*O*-*α*-rhamnopyranosyl-*β*-glucopyranosides) and pelargonidin-3-*O*- (6″-*O*-*α*-rhamnopyranosyl-*β*-glucopyranosides) were detected, with Pg3G existing as a minor component [15], [16].

In this study, we hypothesized that the floral flowers of *L. longituba* are assocated with the components of anthocyanins, a group of water-soluble vacuolar pigments, which can be measured readily by using high-performance liquid chromatography, coupled with a photodiode array detection and electrospray ionization mass spectrometry (HPLC–ESI–MS^n^) analysis.

We measured anthocyanin components in different colors of flowers derived from 44 variants of *L. longituba*. These variants were sampled randomly from a population in the natural distribution of this species. Four major components in the petal extracts, Cy3So, Cy3XyGlc, cyanidin-3-sophoroside (Cy3Sa), and Pg3XyGlc, were identified. Through a multivariate analysis for the correlations between color and anthocyanin contents, we suggested that different amounts of these anthocyanin components are important determinants for the natural variation in flower colors for *L. longituba*.

## Results

### Petal Colors

**Figure 2:**
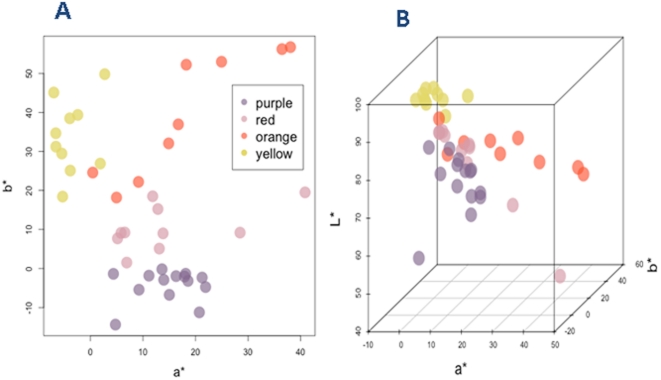
Distribution of *Lycoris longituba* variants ants by flower colors based on a bivariate (a* and b*) (A) and trivariate (a*, b*, and L*) CIELAB data (B), respectively. The flower colors were identified by the RHSCC value.

Of the 44 variants sampled, one has white flowers, which is viewed as an ancestral type of *L. longituba* [17]. Floral colors were quantified by the Royal Horticultural Society Colour Chart (RHSCC) [18], with RHSCC values ranging from 4D to 159C except for the white. A software package called CIE 1976 L*a*b* (CIELAB) was used to measure different aspects of a flower color. CIELAB is a one-color system that takes into account all aspects of the color described by L^*^, a^*^, and b^*^ parameters. The L^*^ describes the lightness of the color, ranging from black (L^*^ = 0) to white (L^*^ = 100). The a^*^ negative and positive are for green and red, and the b* negative and positive are for blue and yellow, respectively [19]. The color coordinates measured were shown as follows: a^*^ values ranged from −6.99 to 40.87, b^*^ values from −14.36 to 56.71, and the L^*^ values from 45.95 to 93.03 (Fig. 2).

Using Tian's approach [20], we derived two new parameters from a^*^ and b^*^, which are Chroma [C^*^ = (a^*^ ^2^+b^*^ ^2^)^1/2^] and hue angle [*h* = arctan b^*^/a^*^ ]. The Chroma parameter describes the saturation of the color, a measure of how far the color is from the grey tone. The higher the C^*^ value, the more saturated is the color. The hue angle parameter describes the hue of the color, i.e. color tonalities (red, yellow, blue, etc.) [21], [22]. A red color has *h* around 0° while yellow is described by an *h* around 90°. Thus, *h* = 45° corresponds to orange [15], [19]. Based on the hue angles measured, 43 variants were classified into four groups in terms of floral colors (Fig. 1), purple (14), red (10), orange (9), and yellow (10) (Table 1). All the groups have a high L^*^ value (except for LL105). However, L^*^ values of the orange group were lower than those of the other groups, with the maximum value of 64.60 in the orange group, compared with 92.69 in the yellow group.

**Table 1:** Petal colors and color parameters of 44 *Lycoris longituba* variants.

Color	RHSCC^y^	*L* ^*y^	*a* ^*y^	*b* ^*y^	*C* ^*y^	*h* ^y^
**Purple group**						
LL39	75D	81.06	13.62	−0.19	13.7	−0.79
LL46	75C	80.31	13.97	−2.89	14.27	−11.7
LL49	75C	78.98	18.57	−3.17	18.87	−9.7
LL50	73B	78.41	17.92	−2.06	18.09	−6.56
LL52	77D	71.68	21.26	−2.32	21.4	−6.22
LL54	75D	78.53	16.3	−1.95	16.44	−6.82
LL55	91C	78.46	9.24	−5.43	10.85	−30.46
LL57	75A	73.31	21.92	−4.75	22.43	−12.24
LL81	77D	71.66	18.13	−1.35	18.2	−4.25
LL86	76C	84.56	4.37	−1.37	4.69	−17.43
LL93	75C	84.3	11.14	−1.83	11.34	−9.34
LL103	104B	58.21	4.76	−14.36	15.36	−71.69
LL24	84C	75.52	15.02	−6.74	16.56	−24.18
LL87	98C	68.98	20.73	−11.23	23.71	−28.46
**Red group**						
LL26	65B	80.81	11.85	18.51	21.99	57.39
LL27	73B	82.34	13.11	5.11	14.1	21.31
LL33	69B	78.05	13.82	9.01	16.6	33.1
LL37	69C	87.81	6.91	1.52	7.27	12.39
LL40	36D	86.47	5.82	9.13	11.02	57.5
LL56	38D	85.35	6.54	9.23	11.37	54.71
LL61	36C	80.97	12.84	15.25	20.04	49.93
LL80	65D	86.5	5.16	7.72	9.37	56.28
LL105	47B	45.95	40.87	19.51	45.3	25.53
LL106	52C	66.98	28.49	9.2	30.01	17.91
**Orange group**						
LL53	24D	80.63	9.15	22.19	24.02	67.61
LL59	159C	86.39	0.43	24.58	24.6	89.03
LL62	27B	78.38	4.96	18.19	18.93	74.77
LL63	31D	74.36	16.73	36.93	40.58	65.66
LL66	28B	66.51	36.48	56.2	67.02	57.04
LL74	29C	78.88	14.89	32.05	35.37	65.12
LL79	24A	75.13	18.24	52.22	55.39	70.78
LL90	28A	64.6	38.09	56.71	68.33	56.14
LL107	26A	68.62	24.96	52.97	58.57	64.8
**Yellow group**						
LL31	12C	88.03	−2.42	39.35	39.43	−86.52
LL34	8C	89.88	−6.99	45.09	45.63	−81.23
LL35	8D	90.19	−5.46	29.45	29.96	−79.54
LL43	50D	90.31	−3.85	25.09	25.39	−81.31
LL44	11C	86.53	1.86	26.88	27.02	86.09
LL45	21C	86.76	2.72	49.79	49.92	86.91
LL51	4D	92.69	−5.31	18.43	19.18	−73.97
LL67	10D	92.01	−6.6	34.65	35.28	−79.26
LL75	10B	89.75	−3.94	38.48	38.69	−84.2
LL77	4D	91.38	−6.58	31.21	31.9	−78.13

a^*^, b^*^, chromatic components; C^*^, chroma; *h*, hue angle (°); L^*^, lightness; RHSCC, Royal Horticultural Society Colour Chart.

### Anthocyanin Identification

**Figure 3:**
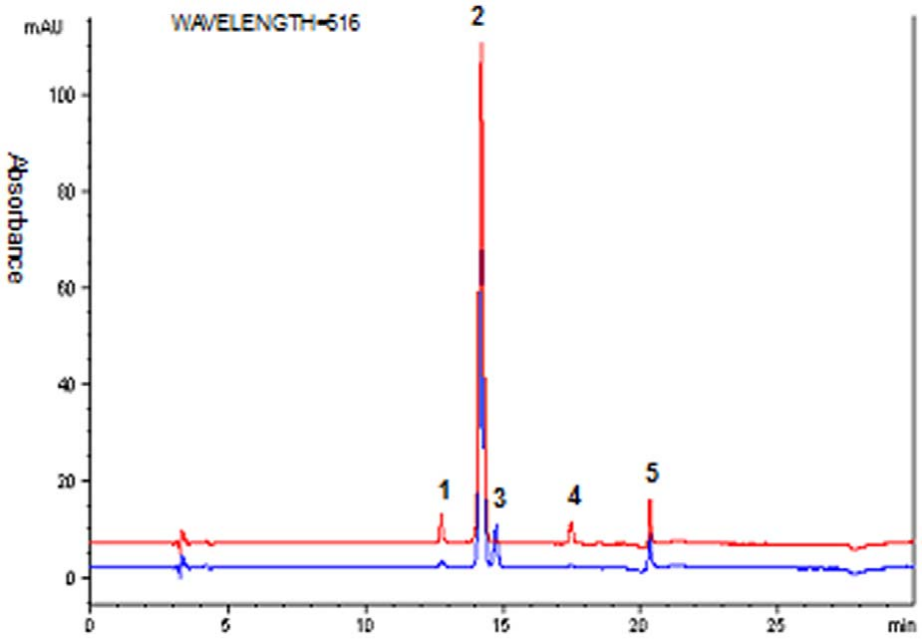
The HPLC chromatogram of *Lycoris longituba* petal extracts detected by light wavelength = 516 nm. Peak labels: (1) cyanidin 3-sophoroside (Cy3So); (2) cyanidin 3-xylosylglucoside) (Cy3XyGlc); (3) cyanidin 3-sambubioside (Cy3Sa); (4) pelargonidin 3- xylosylglucoside (Pg3XyGlc); (5) Unidentified. Two colors, red and blue, denote the HPLC chromatograms of two different *L. longituba* varieties, LL52 and LL27, respectively.

**Figure 4:**
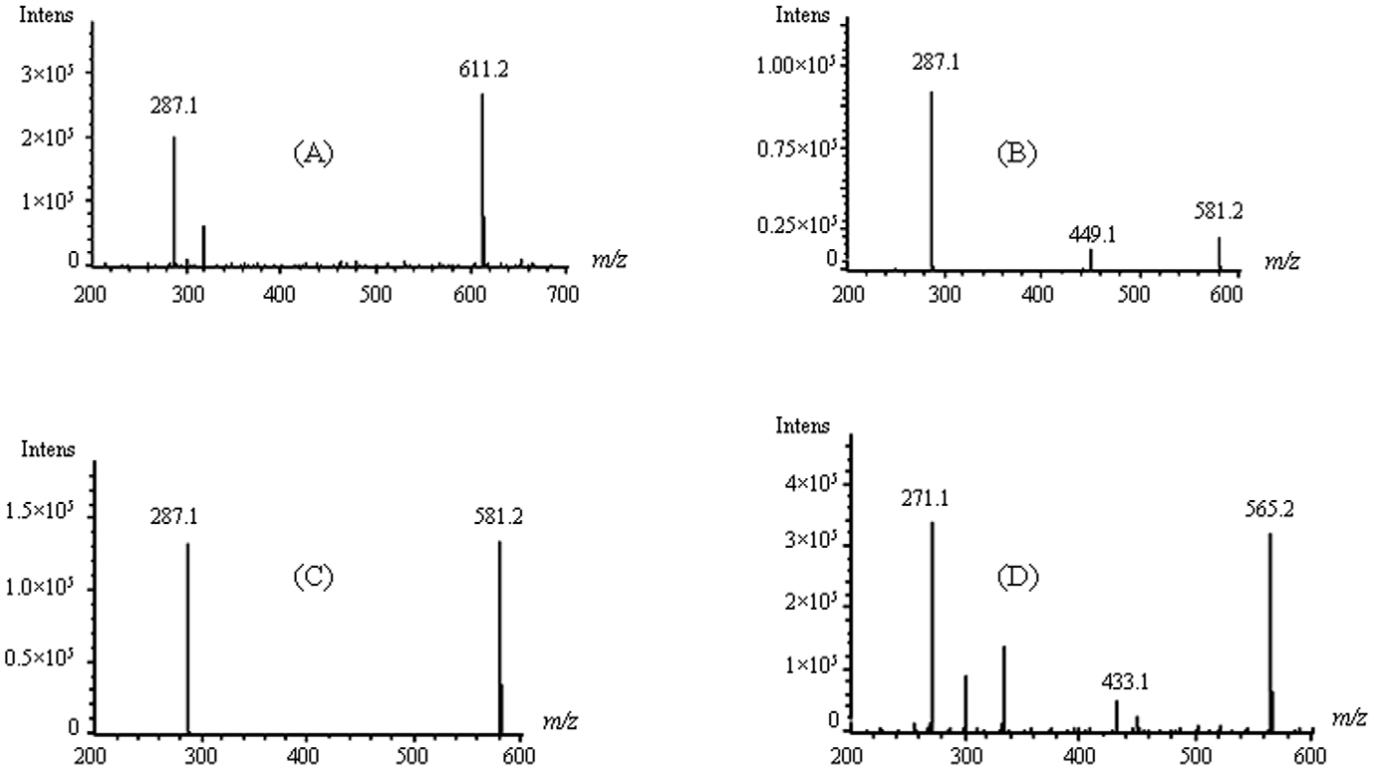
The HPLC–ESI/MS/MS of anthocyanins in *Lycoris longituba*. Mass spectra of anthocyanin components. (A) cyanidin 3-sophoroside, (B) cyanidin 3-xylosylglucoside, (C) cyanidin 3-sambubioside, and (D) pelargonidin 3-xylosylglucoside.

Figure 3 shows an HPLC–ESI–MS^n^ analysis of anthocyanins in *L. longituba* variants. UV–vis detection (by light wavelength 516 nm) showed the presence of two major and three minor anthocyanins. The change of the mobile phase helped the separation of peak 1 from peaks 2, 3, 4 and 5. Chemical structures of anthocyanins were confirmed by HPLC–ESI–MS^n^. The HPLC retention time (Rt), elution order, and their UV–vis spectral properties together with mass spectrometric data were shown in Table 2. Peaks 1, 2, and 3 all produced a major fragment at *m/z* 287, indicating that they were cyanidin (Cy) derivatives (Fig. 3). They exhibited molecular ions at *m/z* 611 and 581. The cyanidin anthocyanin with a molecular ion of *m*/*z* 611 detected by the HPLC–ESI–MS^n^ analysis was identified as cyanidin 3-sophoroside (Fig. 4A), because this compound only produced one major fragment at *m/z* 287 [23]. Similarly, peak 3 was identified as Cy3Sa (Fig. 4C). The molecular ion of *m/z* 581 was fragmented into two major fragments at *m/z* 287 and 449 in peak 2 identified as Cy3XyGlc (Fig. 4B). The minor anthocyanin (*m*/*z* 565, peak 4 identified as Pg3XyGlc in Fig. 2) was identified as a pelargonidin anthocyanin, which was a precursor of *m*/*z* 271 (Fig. 4D). The molecular ion at *m/z* 565 was fragmented at *m/z* 271 and 433. In addition, the identities of Cy3So and Cy3Sa were further confirmed using their respective standards. Peak 5 produced a major fragment at *m/z* 303, but its identity was not confirmed because of sample limitation. The amounts of various components of anthocyanins measured for individual *L. longituba* varieties are given in Table 3.

**Table 2:** Chromatographic and spectral data of anthocyanins detected in 44 *Lycoris longituba* varieties.

Peak	Anthocyanins^a^	t_R_ b (min)	λ^c^ (nm)	Molecular ion	Fragment ions	
				[M+H]^+^(*m*/*z*)		
1	Cy3So	12.712	282.0,514.2	611.2	287.1[M+H-162-162]^+^	
2	Cy3XyGlc	14.157	280.2,516.3	581.2	449.1[M+H-132]^+^	287.1[M+H-132-162]^+^
3	Cy3Sa	14.673	281.0,500.1	581.2	287.1[M+H-162-132]^+^	
4	Pg3XyGlc	20.267	278.0,513.9	565.2	433.1[M+H-132]^+^	271.1[M+H-132-162]^+^

a Cy, cyanidin; Pg, pelargonidin; So, sophorose; XyGlc, xylosylglucoside ; Sa, sambubioside.

b t_R_, retention time.

c λ, absorption wavelength.

**Table 3:** Petal anthocyanin mean values (in peak areas %) of 44 *Lycoris longituba* varieties.

Variety	Cy^a^			Pg^a^	U^a^	Aglycone^a^			Glucside^a^			TA^b^	TF^b^	CI^b^
	3So	3XyGl	3Sa	3XyGl		Cy	Pg	U	3So	3XyGl	3Sa			
**Purple**														
LL39	-	78	12	-	10	90	-	10	-	78	12	0.2168	1.905	8.78
LL46	4	83	8	5	-	95	5	-	4	88	8	0.3223	1.3284	4.12
LL49	-	79	21	-	-	100	-	-	-	79	21	0.1733	0.9844	5.68
LL50	4	83	8	5	-	95	5	-	4	88	8	0.2687	1.3126	4.88
LL52	3	85	-	3	9	88	3	12	3	88	9	0.3086	0.7636	2.28
LL54	-	85	15	-	-	100	-	-	-	85	15	0.1375	1.0848	7.88
LL55	-	81	19	-	-	100	-	-	-	81	19	0.1584	0.7544	4.76
LL57	5	84	11	-	-	100	-	-	5	84	11	0.2226	0.76	3.42
LL81	4	82	14	-	-	100	-	-	4	82	14	0.4066	1.729	4.26
LL86	-	100	-	-	-	100	-	-	-	100	-	0.1059	1.3272	12.52
LL93	-	100	-	-	-	100	-	-	-	100	-	0.1263	1.0802	8.56
LL103	9	79	-	12	-	88	12	-	9	91	-	0.4333	1.3196	3.04
LL24	5	86	-	-	9	91		9	5	86	-	0.2945	0.8366	2.84
LL87	9	78	-	13	-	87	13	-	9	91	-	0.4643	1.5572	3.35
**Red**														
LL26	-	88	12	-	-	100	-	-	-	88	12	0.1889	0.899	4.76
LL27	-	73	16	-	11	89	-	11	-	84	16	0.1271	1.3686	10.77
LL33	-	83	17	-	-	100	-	-	-	83	17	0.1304	1.3562	10.4
LL37	-	74	26	-	-	100	-	-	-	74	26	0.0619	0.9938	16.05
LL40	-	90	10	-	-	100	-	-	-	90	10	0.0751	0.557	7.42
LL56	-	86	14	-	-	100	-	-	-	86	14	0.0981	1.3234	13.49
LL61	-	87	13	-	-	100	-	-	-	87	13	0.1096	0.2344	2.14
LL80	-	91	9	-	-	100	-	-	-	91	9	0.0855	0.4138	4.84
LL105	-	95	5	-	-	100	-	-	-	95	5	1.6703	0.1136	0.07
LL106	-	66	34	-	-	100	-	-	-	66	34	1.2143	0.0884	0.07
**Orange**														
LL53	-	100	-	-	-	100	-	-	-	100	-	0.1007	1.0034	9.96
LL59	-	100	-	-	-	100	-	-	-	100	-	0.0487	0.814	16.71
LL62	4	84	7	5	-	95	5	-	4	89	7	0.2579	0.6958	2.7
LL63	-	88	12	-	-	100	-	-	-	88	12	0.1732	0.9426	5.44
LL66	-	90	10	-	-	100	-	-	-	90	10	0.2112	0.5656	2.68
LL74	-	92	8	-	-	100	-	-	-	92	8	0.1585	0.2256	1.42
LL79	82	13	-	-	5	95	-	5	82	13	5	0.1941	1.1146	5.74
LL90	4	89	7	-	-	100	-	-	4	89	7	0.4703	0.8444	1.8
LL107	-	86	14	-	-	100	-	-	-	86	14	0.2022	0.483	2.38
**Yellow**														
LL31	-	-	-	-	-	-	-	-	-	-	-	-	1.2104	-
LL34	-	-	-	-	-	-	-	-	-	-	-	-	0.2166	-
LL35	-	-	-	-	-	-	-	-	-	-	-	-	0.6768	-
LL43	-	-	-	-	-	-	-	-	-	-	-	-	1.0508	-
LL44	-	-	-	-	-	-	-	-	-	-	-	-	0.875	-
LL45	-	-	-	-	-	-	-	-	-	-	-	-	0.1806	-
LL51	-	-	-	-	-	-	-	-	-	-	-	-	0.7076	-
LL67	-	-	-	-	-	-	-	-	-	-	-	-	0.1708	-
LL75	-	-	-	-	-	-	-	-	-	-	-	-	0.1436	-
LL77	-	-	-	-	-	-	-	-	-	-	-	-	0.5888	-
**White**														
LL38	-	-	-	-	-	-	-	-	-	-	-	-	0.3336	-

a Cy: cyanidin; Pg: pelargonidin; 3So: 3-sophoroside; 3GXyGlc: 3-xylosylglucoside; 3Sa: 3-sambubioside; U: the unidentified fifth anthocyanin(sample limitations did not allow complete confirmation of the identity). Data are expressed as percentage.

b TA: total anthocyanin; TF: total flavones and flavonols in mg per 100 mg dry petals; CI: copigment index = TF/TA.

-: not detected.

### Relationships between Color Parameters and Anthocyanin Compositions

#### Relationship between C^*^ and L^*^ values

**Figure 5:**
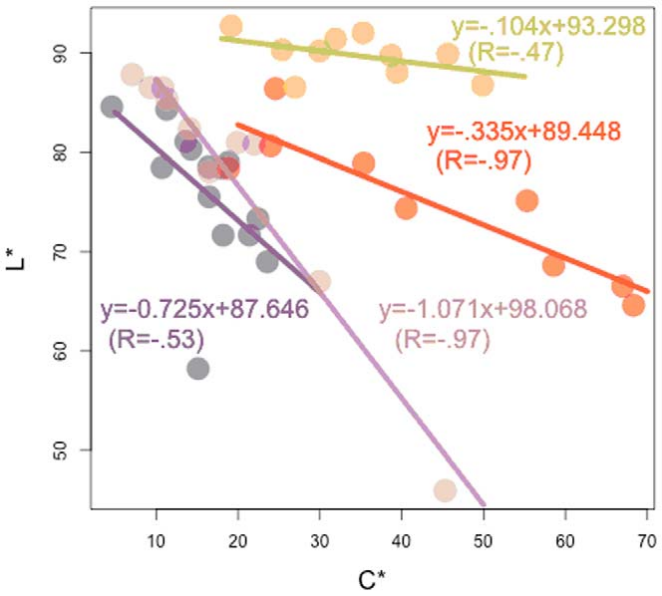
The multivariate relationships between C^*^ and L^*^ values, showing different patterns depending on petal colors, purple, red, orange, and yellow (see Fig. 1), in *Lycoris longituba* flowers.

In all the groups, except for yellow, the L^*^ value was negatively correlated with the C^*^ value for all groups (Fig. 5), with a steeper slope for purple, followed by red, orange and yellow. This suggests that the less bright the flower, the more saturated the flower color.

#### Relationship between L^*^ value and the amount of total anthocyanins (TA)

There was the same tendency of a negative correlation between these two variables in red, purple, and orange groups. The L^*^ value increased as the TA amount decreased. It means that the brighter the flower petals, the less the TA amount. The coefficient of determination (R) of the red group (0.92) was much larger than those of purple (0.64) and orange groups (0.58). In white and yellow groups, the TA amounts were nearly zero.

#### Relationship between a^*^ value and TA

The red and orange groups showed a positive correlation (R = 0.93 and 0.49, respectively), whereas the correlation between a^*^ value and TA was not found in the purple group. In general, the a^*^ value increased as TA increased.

#### Relationship between a^*^ value and CI

The co-pigmentation effect occurs when the TF/TA ratio (co-pigmentation index, CI) exceeds 5 [24]. This phenomenon was observed in all the groups. The red and orange groups showed a negative correlation between a^*^ value and CI. Since the TA values of the white and yellow groups were zero, they were excluded from the CI analysis.

#### Multiple linear regression (MLR) analysis of color parameters and anthocyanin compositions

The MLR analysis chooses the following equations that best fit the relationships between color parameters and anthocyanin components (*p*<0.01):
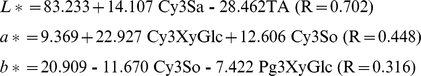



From this MLR analysis, it can be seen that the Cy derivatives were the major factors that affect color parameters. Cy3XyGlc and Cy3So had positive effects on the value of a^*^, whereas Cy3So and Pg3XyGlc had negative effects on the value of b^*^. TA was the key factor that reduces the L^*^ value. In addition, Cy3Sa had a positive effect on the value of L^*^.

## Discussion

*Lycoris longituba*, an annual species, is characteristic of a few unique biological features [1]. For example, a distinct diversity of floral colors pervades its narrow natural distribution. In general, the flower colors of this species are grouped into four types, purple, red, orange, and yellow (Fig. 1). In this study, we studied the natural variation of floral color in *L. longituba* by quantifying the components of anthocyanins in flower petals of each type. As water-soluble vacuolar pigments, anthocyanins are an important phenotype for studying the evolution of floral colors and may be used as an indirect trait to select elite genotypes that are optimally adaptive to changing environment [4]–[6], [9]–[12]. Through tests and comparsions, we suggest that differences in anthocyanin type and quantity were responsible for diverse floral colors of *L. longituba*. This result may not only provide a biochemical basis for floral colors, but also helps to understand the adaptation and evolution of floral colors to changing environment.

By using high-performance liquid chromatography, coupled with photodiode array detection and electrospray ionization mass spectrometry, we identified four major types of anthocyanin, cyanidin-3-sophoroside (Cy3So), cyanidin-3-xylosylglucoside (Cy3XyGlc), cyanidin-3-sambubioside (Cy3Sa), and pelargonidin-3-xylosylglucoside (Pg3XyGlc), in the petal extracts of *L. longituba*. To our best knowledge, Cy3So and Cy3Sa have not been reported in any species of the *Lycoris* genus, but they were found to be an important determinant of floral colors in *L. longituba*. Cy3XyGlc had a wide distribution in purple, red, and orange varieties, occurring in more than 70% variants. Cy3So occurs in all but one sample, accompanied by Pg3XyGlc in the purple group. Furthermore, Pg3XyGlc was only detected in the purple group. But in the red group, no Cy3So was detected. We found that the major flower flavonols of *L. longituba* were 3-xylosylglucoside of kaempferol and quercetin (Km3XyGlc and Qu3XyGlc) (Q. L. He, unpublished data). This suggests that the same glycosylation process occurred for flavonols and anthocyanins.

By estimating and using quantitative color parameters with color measuring systems, we are able to interpret the tremendous variation of floral color in *L. longituba* naturally distributed within a small range. It is suggested from our study that a wide array of floral colors is mainly due to the chemical structures of different anthocyanins accumulated in the flower. The correlations between color parameters, L^*^ and C^*^ (Fig. 5), L^*^ and TA value, a^*^ and CI value, showed negative tendencies in all the groups except for yellow. An inverse tendency was detected between a^*^ and TA in red and orange groups. A correlation analysis indicates that the *L* ^*^ value increased with increasing proportions of Cy3XyGlc and Cy3Sa. These results can be integrated into an evolutionary study to study the ecological and evolutionary relationship of floral colors with environmental factors [2]. In practice, these also help to develop appropriate criteria for breeding favorable colors through indirect selection.

The type and content of anthocyanins are important determinants for flower coloration. All four anthocyanins occur in most variants from the purple group, whereas only three (Cy3So, Cy3XyGlc, and Cy3Sa) exist in some orange variants and only two (Cy3XyGlc and Cy3Sa) exist in all red variants. Thus, the *in vivo* colors of *L. longituba* seem to be correlated with the anthocyanin types in the petals, although there were some exceptions. In some variants, Cy3XyGlc is the sole anthocyanin, but their petals display orange and purple-red colors. In a study of tulips, Nieuwhof et al. [25] found that yellow and orange colors of petals may be due to carotenoids. The petals of some plants have a capacity to modify carotenoid biosynthesis, producing an orange-red color [26]. Astaxanthin is a ketocarotenoid that is produced in a few plants such as *Adonis aestivalis*; it furnishes an attractive orange-red color [27]. The orange petals of calendula (*Calendula officinalis*) contain reddish carotenoids that are absent in yellow petals [28]. These species contain very few types of anthocyanins, and their yellow, purple and orange colors indeed reveal that other factors than anthocyanins (carotenoids, co-pigmentation, pH, etc.) may influence the flower color [3]. However, carotenoids are highly unstable and difficult to extract and measure in practice [29]. Taken together, we suggest that anthocyanins can be practically more useful to explain the floral colors of *L. longituba* and their tremendous diversity in nature and can further be used as an indirect trait to select elite genotypes in flower color.

*L. longituba* has a limited range of distribution, but is characterized by a high diversity of floral colors. A next step for studying this species should be to understand the pattern of genetic diversity using molecular markers and the evolution of phytochrome function operated by anthocyanins [2]. More recently, we have exploited an array of informative microsatellite markers [30] and also developed a series of statistical models for estimating and testing multiallelic Hardy-Weinberg disequilibria and multiallelic linkage disequilibria with multilocus data [31]. By associating with marker polymorphisms with floral colors, we can identify significant genetic variants that contribute to flower variation, shedding light on the genetic architecture of color formation in *L. longituba*.

## Materials and Methods

### Plant Material and Petal Color Measurement

Flowers of 44 *L. longituba* variants were collected from the *Lycoris* plantation of Nanjing Forestry University in Nanjing, China. Three petal samples of each variant were collected at a full-bloom stage from late July to early August in 2008. The 44 variants were divided into four groups according to flower color – purple, red, orange, and yellow.

The colors of fresh petals of the 44 variants were first compared with the Royal Horticultural Society Colour Chart (RHSCC) [17] (Royal Horticultural Society 2001), and the surface color of fresh flowers were also analyzed with a spectrophotometer NF333 (Nippon Denshoku Industries Co. Ltd., Japan) using the CIELAB system at CIE C/2° illumination/viewer condition.

### Quantification of Anthocyanin Components

Petals of each sample (approximate 100–150 mg dry weight) were extracted with 5 mL 70% methanol aqueous solution containing 0.1% HCl (pH 2.08) in a test tube at 4°C in the dark for 24 h and shaken in a Gene-2 vortex (Scientific Industries INC, USA) every 6 h [32]. The liquid was separated from the solid mixture by filtration through a sheet of qualitative filter paper (Hangzhou Xinghua Paper Industry, Zhejiang, China). The filtrate passed through 0.2 µm membrane filters (Membrana, Wuppertal, Germany). This experiment was performed three times for each cultivar and the extracts from each experiment were separately used for identification and quantification of anthocyanins.

Chromatographic analysis was carried out on a HPLC (Agilent Corporation, Waldbronn, Germany) equipped with a G1311A HPLC Pump, Thermostatted Column Compartment G1316A and a Diode-Array G1315B Detector. The HPLC column was ZORBAX Eclipse XDB-C18 (250 mm×4.6 mm i.d.; 5 µm) (Agilent Corporation, Sunnyvale, CA, USA) and was protected with a Micro-Guard Cation-H Cartridge (Bio-RAD Laboratories, Hercules, CA, USA). A volume of 10 µL sample solution was injected for HPLC analysis. Flow rate was 0.8 mL/min and the mobile phase A consisted of 10% (v/v) formic acid in water (10∶90; v/v, HCOOH∶H_2_O), while CH_3_CN∶CH_3_OH (85∶15, v/v) was used as the mobile phase B [33]. The column was eluted at 25°C using linear gradients which was 5% B at 0 min, 15% B at 15 min and 90% B for another 6 min. Data were recorded on a computer with the ChemStation software (Agilent Corporation, Waldbronn, Germany). Detection was carried out at 516 nm for anthocyanins and 350 nm for flavones and flavonols, the photodiode array spectra were recorded between 200 and 800 nm. All determinations were performed with three replicates.

Anthocyanins were identified by HPLC–ESI–MS^n^ (Agilent Technologies, Palo Alto, CA, USA). The interface was an ESI–electrospray (Agilent 1100 LC/MSD Trap VL) operating in positive ionization mode monitoring of the protonated and deprotonated molecular ions at the following operating parameters: the operating parameters in positive mode were as follows: gas (N_2_) temperature, 350°C; flow rate, 6.0 L/min; nebulizer pressure, 241.3 kPa; HV voltage, 3.5 kV; octopole RF amplitude, 150 Vpp; skim 1 voltage, 55.6 V; skim 2 voltage, 6.0 V; capillary exit, 140 V; cap exit offset, 84.4 V, and scan range, *m/z* 100–1000 units.

The amount of total anthocyanins (TA) and total flavones and flavonols (TF) were calculated in milligrams per 100 mg dry weight (as a quantity of Mv3G5G mg/100 mg, and as a quantity of Rutin mg/100 mg, respectively). Co-pigmentation index (CI) was calculated by TF/TA [34].

### Statistical Analysis

Correlations between petal color parameters and anthocyanin compositions of individual varieties were analyzed using a multiple linear regression (MLR) (SAS 8.1 software). Color parameters including L^*^, a^*^, and b^*^ values were considered as dependent variables, and the contents of individual anthocyanins and the total anthocyanin content were considered as independent variables.
